# [μ-3,3′-Bis(tri­hydro­bor­yl)[3]ferroceno­phane]bis­(chlorido­zirconocene)

**DOI:** 10.1107/S1600536813023933

**Published:** 2013-09-07

**Authors:** Adelina Reichert, Hans-Wolfram Lerner, Matthias Wagner, Michael Bolte

**Affiliations:** a Institut für Anorganische Chemie, J. W. Goethe-Universität Frankfurt, Max-von-Laue-Strasse 7, 60438 Frankfurt/Main, Germany

## Abstract

The title compound, [FeZr_2_(C_5_H_5_)_4_Cl_2_(C_13_H_18_B_2_)], is a heteronuclear complex that consists of a [3]ferrocenophane moiety substituted at each cyclo­penta­dienyl (Cp) ring by a BH_3_ group; the BH_3_ group is bonded *via* two H atoms to the Zr atom of the zirconocene chloride moiety in a bidentate fashion. The two Cp rings of the [3]ferrocenophane moiety are aligned at a dihedral angle of 8.9 (4)° arising from the strain of the propane-1,3-diyl bridge linking the two Cp rings. [One methyl­ene group is disordered over two positions with a site-occupation factor of 0.552 (18) for the major occupied site.] The dihedral angles between the Cp rings at the two Zr atoms are 50.0 (3) and 51.7 (3)°. The bonding Zr⋯H distances are in the range 1.89 (7)–2.14 (7) Å. As the two Cp rings of the ferrocene unit are connected by an *ansa* bridge, the two Zr atoms approach each other at 6.485 (1) Å. The crystal packing features C—H⋯Cl inter­actions.

## Related literature

For Zr⋯B distances, see: Edelstein (1981 ▶). For information about the coordination behaviour of mono- and ditopic ferrocenyl­hydro­borates toward [Cp_2_ZrCl]^−^, see: Reichert *et al.* (2013*a* ▶). For synthetic details, see: Reichert (2013 ▶); Reichert *et al.* (2013*b* ▶).
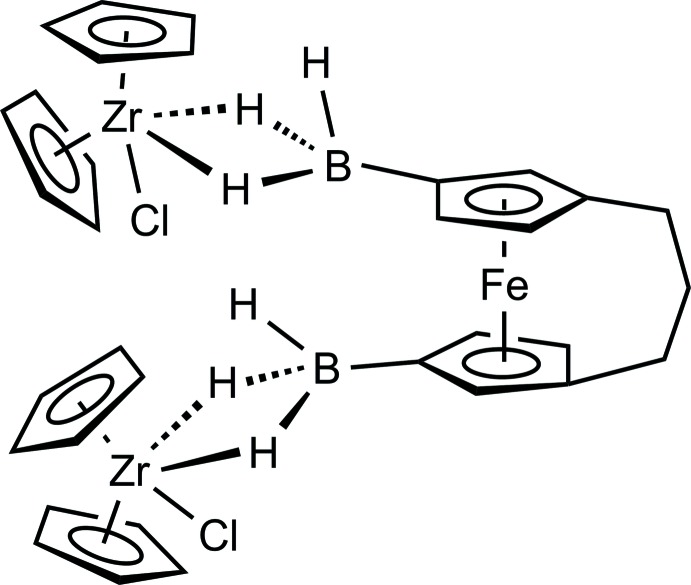


## Experimental

### 

#### Crystal data

[FeZr_2_(C_5_H_5_)_4_Cl_2_(C_13_H_18_B_2_)]
*M* *_r_* = 765.44
Monoclinic, 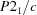

*a* = 18.2954 (9) Å
*b* = 11.6004 (6) Å
*c* = 15.3351 (6) Å
β = 104.963 (3)°
*V* = 3144.3 (3) Å^3^
*Z* = 4
Mo *K*α radiation
μ = 1.30 mm^−1^
*T* = 173 K
0.19 × 0.14 × 0.13 mm

#### Data collection

Stoe IPDS II two-circle diffractometer
Absorption correction: multi-scan (*MULABS*; Spek, 2009 ▶; Blessing, 1995 ▶) *T*_min_ = 0.791, *T*_max_ = 0.849
29348 measured reflections
5885 independent reflections
3926 reflections with *I* > 2σ(*I*)
*R*_int_ = 0.102

#### Refinement

*R*[*F* ^2^ > 2σ(*F* ^2^)] = 0.048
*wR*(*F* ^2^) = 0.112
*S* = 0.94
5885 reflections
379 parameters
H atoms treated by a mixture of independent and constrained refinement
Δρ_max_ = 0.60 e Å^−3^
Δρ_min_ = −1.22 e Å^−3^

### 

Data collection: *X-AREA* (Stoe & Cie, 2001 ▶); cell refinement: *X-AREA*; data reduction: *X-AREA*; program(s) used to solve structure: *SHELXS97* (Sheldrick, 2008 ▶); program(s) used to refine structure: *SHELXL97* (Sheldrick, 2008 ▶); molecular graphics: *XP* in *SHELXTL* (Sheldrick, 2008 ▶); software used to prepare material for publication: *SHELXL97* and *publCIF* (Westrip, 2010 ▶).

## Supplementary Material

Crystal structure: contains datablock(s) I, global. DOI: 10.1107/S1600536813023933/ng5341sup1.cif

Structure factors: contains datablock(s) I. DOI: 10.1107/S1600536813023933/ng5341Isup2.hkl

Additional supplementary materials:  crystallographic information; 3D view; checkCIF report

## Figures and Tables

**Table 1:** Selected bond lengths (Å)

Zr1—Cl1	2.506 (2)
Zr1—B1	2.551 (6)
Zr2—Cl2	2.4616 (17)
Zr2—B2	2.593 (7)

**Table 2:** Hydrogen-bond geometry (Å, °)

*D*—H⋯*A*	*D*—H	H⋯*A*	*D*⋯*A*	*D*—H⋯*A*
C25—H25⋯Cl1^i^	0.95	2.75	3.466 (7)	133
C28—H28⋯Cl1^ii^	0.95	2.80	3.496 (8)	131
C30—H30⋯Cl2	0.95	2.82	3.623 (7)	143

Symmetry codes: (i) 
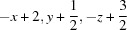
; (ii) 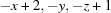.

**Fig. 1.**
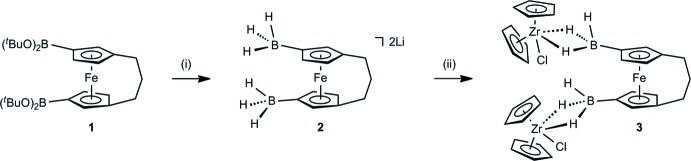
Synthesis of the title compound 3. Reagents and conditions: (i) 2 eq Li[AlH4], Et2O, -78°C to room temperature, 1.5 h; (ii) 3 eq [Cp2ZrCl2], Et2O, room temperature, 12 h.

**Fig. 2.**
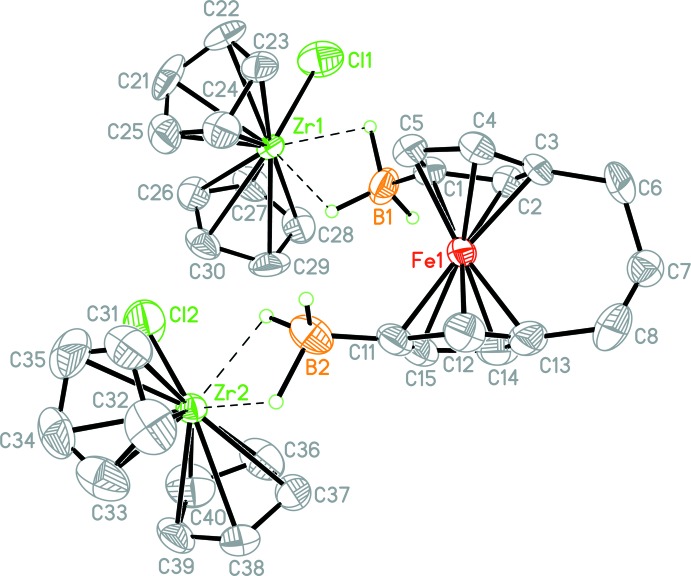
Perspective view of the title compound 3. Displacement ellipsoids are drawn at the 50% probability level. H atoms bonded to C and the minor occupied site of the disordered methylene group omitted for clarity.

## supplementary crystallographic information

### 1. Comment

Recently we have reported on coordination behaviour of mono- and ditopic ferrocenylhydroborates Li[FcBH_3_] and Li_2_[fc(BH_3_)_2_] toward the [Cp_2_ZrCl]^-^ moiety (Fc = ferrocenyl, fc = 1,1'-ferrocenylene, Cp = cyclopentadienyl) (Reichert *et al.*, 2013*a*). Herein we describe the coordination behaviour of ditopic ferrocenylhydroborate derivative 2 that features an ansa bridge in its ferrocene backbone. The title compound 3 was prepared in a two-step one-pot procedure as shown in Fig. 1. The starting material 1 was synthesized according to a previously reported procedure (Reichert *et al.*, 2013*b*).

The title compound 3 (Fig. 2) features a heterotrinuclear complex. It contains a [3]ferrocenophane moiety substituted at the cyclopentadienyl rings with two BH_3_ groups, which are each bonded *via* two H atoms to the Zr centres of the zirconocene chloride moieties. The Zr···B distances show that the two BH_3_ residues coordinate in a bidentate fashion to the Zr centres (Edelstein, 1981).

The two cyclopentadienyl rings of the [3]ferrocenophane moiety are not exactly planar but enclose a dihedral angle of 8.9 (4)° due to the strain of the 1,3-propanediyl bridge linking the two cyclopentadienyl rings. The dihedral angles between the cyclopentadienyl rings at the two Zr centres show almost the same values: 50.0 (3)° and 51.7 (3)°. The bonding Zr···H distances are in the range from 1.89 (7) Å to 2.14 (7) Å and the H atoms of the BH_3_ groups not bonded to Zr show a H···Zr distance of 3.14 (7) Å and 3.13 (8) Å. Since the two cyclopentadienyl rings of the ferrocene unit are connected by an ansa bridge, the two Zr centres approach each other to a distance of 6.485 (1) Å compared to 7.128 (1) Å in the compound without an ansa bridge (Reichert *et al.*, 2013*a*). The crystal packing is stabilized by C—H···Cl interactions.

### 2. Experimental

All reactions and manipulations were carried out under dry nitrogen or argon with carefully dried and degassed solvents, flame-dried glassware and Schlenk or glove-box techniques. Benzene and Et_2_O were dried over Na/benzophenone and distilled prior to use.

A solution of Li[AlH_4_] in Et_2_O (1 *M*, 0.86 ml, 0.86 mmol) was diluted with Et_2_O (5 ml) and added dropwise with stirring at -78°C to a solution of 1 (0.242 g, 0.449 mmol) in Et_2_O (15 ml). After 30 min, the mixture was allowed to warm to room temperature and stirred for another 1 h. The insolubles were removed by filtration (G4 frit) and washed with Et_2_O (2 × 5 ml). The solvent was removed from the filtrate under reduced pressure and the solid residue was further dried under dynamic vacuum overnight to obtain 2^.^(Et_2_O) as a yellow compound; the amount of Et_2_O present in the sample after drying was determined by ^1^H NMR spectroscopy. Afterwards, in a glove box, neat solid 2^.^(Et_2_O) (0.113 g, 0.333 mmol) and [Cp_2_ZrCl_2_] (0.292 g, 0.999 mmol) were charged to a flask and suspended in Et_2_O (30 ml). The suspension was stirred at room temperature overnight, the red insoluble material was isolated by filtration, washed with Et_2_O (3 × 10 ml) and treated with benzene. LiCl was removed by filtration and the filtrate was slowly evaporated to grow single crystals suitable for X-ray diffraction.

### 3. Refinement

One methylene group is disordered over two positions with a site occupation factor of 0.552 (18) for the major occupied site. The disordered atoms were isotropically refined. H atoms were located in a difference map, but those bonded to C were geometrically positioned and refined using a riding model with fixed individual displacement parameters [U(H) = 1.2 *U*_eq_(C)] and with C_aromatic_—H = 0.95 Å and C_methylene_—H = 0.99 Å. The H atoms bonded to B were freely refined.

### Crystal data

**Table:**

[FeZr_2_(C_5_H_5_)_4_Cl_2_(C_33_H_38_B_2_)]	*F*(000) = 1544
*M**_r_* = 765.44	*D*_x_ = 1.617 Mg m^−^^3^
Monoclinic, *P*2_1_/*c*	Mo *K*α radiation, λ = 0.71073 Å
Hall symbol: -P 2ybc	Cell parameters from 14288 reflections
*a* = 18.2954 (9) Å	θ = 3.3–25.9°
*b* = 11.6004 (6) Å	µ = 1.30 mm^−^^1^
*c* = 15.3351 (6) Å	*T* = 173 K
β = 104.963 (3)°	Block, red
*V* = 3144.3 (3) Å^3^	0.19 × 0.14 × 0.13 mm
*Z* = 4	

### Data collection

**Table:**

Stoe IPDS II two-circle diffractometer	5885 independent reflections
Radiation source: fine-focus sealed tube	3926 reflections with *I* > 2σ(*I*)
Graphite monochromator	*R*_int_ = 0.102
ω scans	θ_max_ = 25.6°, θ_min_ = 3.2°
Absorption correction: multi-scan (*MULABS*; Spek, 2009; Blessing, 1995)	*h* = −22→21
*T*_min_ = 0.791, *T*_max_ = 0.849	*k* = −13→14
29348 measured reflections	*l* = −18→18

### Refinement

**Table:**

Refinement on *F*^2^	Primary atom site location: structure-invariant direct methods
Least-squares matrix: full	Secondary atom site location: difference Fourier map
*R*[*F*^2^ > 2σ(*F*^2^)] = 0.048	Hydrogen site location: inferred from neighbouring sites
*wR*(*F*^2^) = 0.112	H atoms treated by a mixture of independent and constrained refinement
*S* = 0.94	*w* = 1/[σ^2^(*F*_o_^2^) + (0.0552*P*)^2^] where *P* = (*F*_o_^2^ + 2*F*_c_^2^)/3
5885 reflections	(Δ/σ)_max_ = 0.001
379 parameters	Δρ_max_ = 0.60 e Å^−^^3^
0 restraints	Δρ_min_ = −1.22 e Å^−^^3^

### Special details

**Table:**

Geometry. All e.s.d.'s (except the e.s.d. in the dihedral angle between two l.s. planes) are estimated using the full covariance matrix. The cell e.s.d.'s are taken into account individually in the estimation of e.s.d.'s in distances, angles and torsion angles; correlations between e.s.d.'s in cell parameters are only used when they are defined by crystal symmetry. An approximate (isotropic) treatment of cell e.s.d.'s is used for estimating e.s.d.'s involving l.s. planes.
Refinement. Refinement of *F*^2^ against ALL reflections. The weighted *R*-factor *wR* and goodness of fit *S* are based on *F*^2^, conventional *R*-factors *R* are based on *F*, with *F* set to zero for negative *F*^2^. The threshold expression of *F*^2^ > σ(*F*^2^) is used only for calculating *R*-factors(gt) *etc*. and is not relevant to the choice of reflections for refinement. *R*-factors based on *F*^2^ are statistically about twice as large as those based on *F*, and *R*- factors based on ALL data will be even larger.

### Fractional atomic coordinates and isotropic or equivalent isotropic displacement parameters (Å^2^)

**Table:**

	*x*	*y*	*z*	*U*_iso_*/*U*_eq_	Occ. (<1)
Fe1	0.62987 (4)	0.20526 (7)	0.41160 (5)	0.0350 (2)	
Zr1	0.90928 (3)	0.13840 (5)	0.64114 (3)	0.02916 (14)	
Zr2	0.74182 (3)	0.63285 (5)	0.54861 (3)	0.03069 (14)	
Cl1	0.94734 (12)	−0.06969 (18)	0.65470 (13)	0.0694 (5)	
Cl2	0.85626 (11)	0.53838 (18)	0.64097 (14)	0.0748 (6)	
B1	0.7968 (4)	0.1121 (6)	0.5043 (5)	0.0472 (18)	
H1A	0.815 (4)	0.199 (7)	0.538 (5)	0.071*	
H1B	0.838 (4)	0.027 (6)	0.560 (5)	0.071*	
H1C	0.818 (4)	0.101 (7)	0.440 (5)	0.071*	
B2	0.6491 (4)	0.4611 (7)	0.5031 (6)	0.0507 (18)	
H2A	0.719 (4)	0.455 (7)	0.546 (5)	0.076*	
H2B	0.644 (4)	0.577 (7)	0.491 (5)	0.076*	
H2C	0.619 (4)	0.442 (7)	0.541 (5)	0.076*	
C1	0.7094 (3)	0.0939 (5)	0.4866 (4)	0.0404 (13)	
C2	0.6616 (4)	0.0409 (5)	0.4059 (4)	0.0463 (15)	
H2	0.6799	0.0073	0.3591	0.056*	
C3	0.5857 (4)	0.0458 (5)	0.4059 (4)	0.0510 (17)	
C4	0.5817 (4)	0.1002 (5)	0.4880 (4)	0.0497 (16)	
H4	0.5371	0.1142	0.5071	0.060*	
C5	0.6573 (4)	0.1299 (6)	0.5364 (4)	0.0478 (15)	
H5	0.6708	0.1679	0.5932	0.057*	
C11	0.6313 (3)	0.3837 (5)	0.4171 (4)	0.0414 (13)	
C12	0.5578 (3)	0.3396 (5)	0.3733 (5)	0.0493 (16)	
H12	0.5131	0.3544	0.3920	0.059*	
C13	0.5614 (4)	0.2710 (6)	0.2983 (4)	0.0510 (17)	
C14	0.6383 (4)	0.2727 (6)	0.2925 (4)	0.0509 (16)	
H14	0.6577	0.2357	0.2480	0.061*	
C15	0.6803 (3)	0.3407 (5)	0.3666 (4)	0.0454 (15)	
H15	0.7332	0.3549	0.3799	0.054*	
C6	0.5191 (5)	0.0112 (7)	0.3296 (5)	0.076 (3)	
H6A	0.4723	0.0188	0.3503	0.091*	0.552 (18)
H6B	0.5245	−0.0710	0.3152	0.091*	0.552 (18)
H6C	0.5393	−0.0273	0.2832	0.091*	0.448 (18)
H6D	0.4900	−0.0475	0.3535	0.091*	0.448 (18)
C7	0.5098 (7)	0.0846 (10)	0.2403 (7)	0.044 (3)*	0.552 (18)
H7A	0.5553	0.0701	0.2185	0.052*	0.552 (18)
H7B	0.4664	0.0514	0.1947	0.052*	0.552 (18)
C7'	0.4720 (9)	0.0881 (14)	0.2894 (11)	0.053 (5)*	0.448 (18)
H7C	0.4470	0.1191	0.3344	0.064*	0.448 (18)
H7D	0.4324	0.0477	0.2432	0.064*	0.448 (18)
C8	0.4994 (4)	0.2003 (8)	0.2388 (5)	0.074 (2)	
H8A	0.4519	0.2164	0.2564	0.088*	0.552 (18)
H8B	0.4920	0.2272	0.1759	0.088*	0.552 (18)
H8C	0.5168	0.1723	0.1867	0.088*	0.448 (18)
H8D	0.4551	0.2508	0.2151	0.088*	0.448 (18)
C21	0.9321 (4)	0.1654 (8)	0.8051 (4)	0.062 (2)	
H21	0.9834	0.1630	0.8392	0.074*	
C22	0.8821 (4)	0.0715 (7)	0.7846 (4)	0.0510 (16)	
H22	0.8932	−0.0056	0.8045	0.061*	
C23	0.8130 (3)	0.1109 (5)	0.7298 (4)	0.0429 (14)	
H23	0.7697	0.0650	0.7049	0.051*	
C24	0.8192 (4)	0.2310 (6)	0.7183 (4)	0.0478 (15)	
H24	0.7810	0.2805	0.6843	0.057*	
C25	0.8920 (4)	0.2636 (6)	0.7660 (4)	0.0544 (17)	
H25	0.9112	0.3400	0.7711	0.065*	
C26	1.0370 (3)	0.2285 (6)	0.6700 (4)	0.0486 (16)	
H26	1.0658	0.2343	0.7310	0.058*	
C27	1.0387 (3)	0.1379 (6)	0.6119 (5)	0.0507 (15)	
H27	1.0700	0.0715	0.6260	0.061*	
C28	0.9866 (4)	0.1599 (6)	0.5287 (5)	0.0543 (17)	
H28	0.9752	0.1107	0.4775	0.065*	
C29	0.9545 (4)	0.2679 (6)	0.5355 (4)	0.0565 (18)	
H29	0.9182	0.3065	0.4892	0.068*	
C30	0.9861 (4)	0.3089 (6)	0.6236 (4)	0.0506 (16)	
H30	0.9743	0.3802	0.6471	0.061*	
C31	0.6712 (5)	0.6539 (6)	0.6671 (5)	0.0599 (19)	
H31	0.6483	0.5869	0.6840	0.072*	
C32	0.6367 (4)	0.7307 (7)	0.5973 (6)	0.065 (2)	
H32	0.5869	0.7255	0.5589	0.078*	
C33	0.6899 (5)	0.8156 (6)	0.5957 (6)	0.065 (2)	
H33	0.6822	0.8803	0.5563	0.078*	
C34	0.7564 (5)	0.7906 (6)	0.6611 (5)	0.0612 (19)	
H34	0.8022	0.8331	0.6724	0.073*	
C35	0.7435 (5)	0.6919 (8)	0.7068 (5)	0.071 (2)	
H35	0.7785	0.6569	0.7565	0.085*	
C36	0.7990 (6)	0.5965 (7)	0.4204 (5)	0.068 (2)	
H36	0.8200	0.5226	0.4158	0.082*	
C37	0.7257 (6)	0.6309 (7)	0.3819 (4)	0.071 (2)	
H37	0.6874	0.5842	0.3447	0.085*	
C38	0.7165 (5)	0.7453 (6)	0.4061 (4)	0.061 (2)	
H38	0.6710	0.7888	0.3898	0.074*	
C39	0.7860 (4)	0.7838 (6)	0.4584 (4)	0.0523 (17)	
H39	0.7967	0.8589	0.4830	0.063*	
C40	0.8371 (5)	0.6932 (8)	0.4683 (6)	0.070 (2)	
H40	0.8887	0.6954	0.5014	0.084*	

### Atomic displacement parameters (Å^2^)

**Table:**

	*U*^11^	*U*^22^	*U*^33^	*U*^12^	*U*^13^	*U*^23^
Fe1	0.0310 (4)	0.0333 (4)	0.0356 (4)	−0.0095 (3)	−0.0009 (3)	0.0049 (3)
Zr1	0.0326 (3)	0.0317 (3)	0.0230 (2)	0.0021 (2)	0.00692 (19)	0.0034 (2)
Zr2	0.0335 (3)	0.0302 (3)	0.0306 (3)	−0.0038 (2)	0.0124 (2)	0.0005 (2)
Cl1	0.0720 (12)	0.0670 (12)	0.0716 (12)	0.0177 (10)	0.0230 (9)	0.0177 (9)
Cl2	0.0657 (12)	0.0649 (12)	0.0770 (12)	0.0187 (9)	−0.0121 (10)	−0.0046 (10)
B1	0.049 (4)	0.049 (4)	0.035 (3)	0.005 (3)	−0.004 (3)	−0.003 (3)
B2	0.044 (4)	0.046 (4)	0.069 (5)	−0.015 (3)	0.027 (4)	0.003 (4)
C1	0.046 (3)	0.034 (3)	0.037 (3)	0.000 (3)	0.002 (3)	0.007 (2)
C2	0.060 (4)	0.033 (3)	0.038 (3)	−0.004 (3)	0.000 (3)	0.002 (3)
C3	0.058 (4)	0.038 (3)	0.048 (4)	−0.025 (3)	−0.003 (3)	0.007 (3)
C4	0.055 (4)	0.046 (4)	0.047 (4)	−0.020 (3)	0.011 (3)	0.007 (3)
C5	0.053 (3)	0.047 (3)	0.037 (3)	−0.008 (3)	0.000 (3)	0.008 (3)
C11	0.039 (3)	0.031 (3)	0.052 (3)	−0.007 (2)	0.009 (3)	0.006 (2)
C12	0.037 (3)	0.049 (4)	0.061 (4)	0.003 (3)	0.010 (3)	0.006 (3)
C13	0.042 (3)	0.049 (4)	0.053 (4)	0.001 (3)	−0.004 (3)	0.015 (3)
C14	0.060 (4)	0.044 (4)	0.049 (4)	0.000 (3)	0.015 (3)	0.017 (3)
C15	0.042 (3)	0.036 (3)	0.058 (4)	−0.007 (3)	0.013 (3)	0.008 (3)
C6	0.084 (5)	0.072 (5)	0.061 (4)	−0.057 (5)	0.000 (4)	−0.002 (4)
C8	0.054 (4)	0.090 (6)	0.057 (4)	−0.003 (4)	−0.024 (4)	0.012 (4)
C21	0.057 (4)	0.109 (7)	0.021 (3)	−0.006 (4)	0.012 (3)	−0.013 (3)
C22	0.061 (4)	0.069 (4)	0.027 (3)	0.010 (4)	0.021 (3)	0.017 (3)
C23	0.046 (3)	0.050 (4)	0.038 (3)	−0.005 (3)	0.021 (3)	0.005 (3)
C24	0.055 (4)	0.047 (4)	0.049 (4)	0.004 (3)	0.029 (3)	−0.003 (3)
C25	0.073 (5)	0.051 (4)	0.050 (4)	−0.012 (4)	0.036 (3)	−0.018 (3)
C26	0.040 (3)	0.064 (4)	0.045 (3)	−0.015 (3)	0.017 (3)	−0.001 (3)
C27	0.043 (3)	0.049 (4)	0.070 (4)	−0.003 (3)	0.032 (3)	−0.003 (3)
C28	0.063 (4)	0.059 (4)	0.053 (4)	−0.020 (3)	0.037 (3)	−0.013 (3)
C29	0.069 (4)	0.059 (4)	0.046 (4)	−0.005 (3)	0.022 (3)	0.024 (3)
C30	0.070 (4)	0.036 (3)	0.059 (4)	−0.015 (3)	0.041 (3)	−0.006 (3)
C31	0.084 (5)	0.048 (4)	0.066 (4)	−0.004 (4)	0.052 (4)	−0.005 (3)
C32	0.054 (4)	0.069 (5)	0.084 (5)	0.009 (4)	0.038 (4)	−0.012 (4)
C33	0.084 (5)	0.042 (4)	0.087 (5)	0.012 (4)	0.053 (5)	−0.001 (4)
C34	0.076 (5)	0.051 (4)	0.067 (5)	−0.009 (4)	0.036 (4)	−0.027 (4)
C35	0.099 (6)	0.083 (6)	0.037 (4)	0.007 (5)	0.028 (4)	−0.013 (4)
C36	0.115 (7)	0.049 (4)	0.067 (5)	−0.014 (4)	0.071 (5)	−0.013 (4)
C37	0.132 (8)	0.054 (4)	0.030 (3)	−0.033 (5)	0.027 (4)	−0.002 (3)
C38	0.095 (6)	0.046 (4)	0.040 (4)	−0.010 (4)	0.011 (4)	0.012 (3)
C39	0.077 (5)	0.034 (3)	0.054 (4)	−0.020 (3)	0.033 (3)	−0.002 (3)
C40	0.060 (4)	0.081 (6)	0.082 (5)	−0.021 (4)	0.042 (4)	0.009 (5)

### Geometric parameters (Å, º)

**Table:**

Fe1—C2	2.001 (6)	C13—C8	1.503 (9)
Fe1—C13	2.011 (6)	C14—C15	1.433 (9)
Fe1—C3	2.012 (6)	C14—H14	0.9500
Fe1—C12	2.028 (6)	C15—H15	0.9500
Fe1—C14	2.029 (6)	C6—C7'	1.282 (16)
Fe1—C15	2.031 (6)	C6—C7	1.583 (13)
Fe1—C4	2.041 (6)	C6—H6A	0.9900
Fe1—C5	2.045 (6)	C6—H6B	0.9900
Fe1—C1	2.061 (5)	C6—H6C	0.9900
Fe1—C11	2.071 (6)	C6—H6D	0.9900
Zr1—C21	2.461 (6)	C7—C8	1.356 (14)
Zr1—C30	2.481 (6)	C7—H7A	0.9900
Zr1—C25	2.488 (6)	C7—H7B	0.9900
Zr1—C26	2.493 (6)	C7'—C8	1.658 (18)
Zr1—C22	2.501 (6)	C7'—H7C	0.9900
Zr1—C29	2.502 (6)	C7'—H7D	0.9900
Zr1—C24	2.505 (6)	C8—H8A	0.9900
Zr1—Cl1	2.506 (2)	C8—H8B	0.9900
Zr1—C23	2.508 (6)	C8—H8C	0.9900
Zr1—C28	2.509 (6)	C8—H8D	0.9901
Zr1—C27	2.521 (6)	C21—C25	1.403 (10)
Zr1—B1	2.551 (6)	C21—C22	1.405 (10)
Zr1—H1A	2.14 (7)	C21—H21	0.9500
Zr1—H1B	2.02 (7)	C22—C23	1.402 (9)
Zr2—Cl2	2.4616 (17)	C22—H22	0.9500
Zr2—C40	2.480 (7)	C23—C24	1.413 (9)
Zr2—C34	2.481 (6)	C23—H23	0.9500
Zr2—C38	2.484 (6)	C24—C25	1.395 (9)
Zr2—C36	2.490 (7)	C24—H24	0.9500
Zr2—C39	2.492 (6)	C25—H25	0.9500
Zr2—C37	2.497 (6)	C26—C30	1.379 (9)
Zr2—C31	2.499 (7)	C26—C27	1.383 (9)
Zr2—C33	2.504 (7)	C26—H26	0.9500
Zr2—C32	2.507 (7)	C27—C28	1.405 (10)
Zr2—C35	2.513 (7)	C27—H27	0.9500
Zr2—B2	2.593 (7)	C28—C29	1.398 (10)
Zr2—H2A	2.11 (8)	C28—H28	0.9500
Zr2—H2B	1.89 (7)	C29—C30	1.408 (10)
B1—C1	1.564 (10)	C29—H29	0.9500
B1—H1A	1.14 (8)	C30—H30	0.9500
B1—H1B	1.40 (7)	C31—C35	1.379 (11)
B1—H1C	1.16 (8)	C31—C32	1.411 (11)
B2—C11	1.559 (11)	C31—H31	0.9500
B2—H2A	1.28 (7)	C32—C33	1.389 (11)
B2—H2B	1.35 (8)	C32—H32	0.9500
B2—H2C	0.92 (8)	C33—C34	1.392 (11)
C1—C5	1.430 (9)	C33—H33	0.9500
C1—C2	1.455 (8)	C34—C35	1.394 (11)
C2—C3	1.389 (10)	C34—H34	0.9500
C2—H2	0.9500	C35—H35	0.9500
C3—C4	1.428 (10)	C36—C37	1.377 (12)
C3—C6	1.510 (8)	C36—C40	1.420 (11)
C4—C5	1.432 (8)	C36—H36	0.9500
C4—H4	0.9500	C37—C38	1.401 (11)
C5—H5	0.9500	C37—H37	0.9500
C11—C15	1.417 (9)	C38—C39	1.391 (10)
C11—C12	1.434 (8)	C38—H38	0.9500
C12—C13	1.415 (10)	C39—C40	1.389 (11)
C12—H12	0.9500	C39—H39	0.9500
C13—C14	1.431 (10)	C40—H40	0.9500
			
C2—Fe1—C13	116.5 (3)	Zr2—B2—H2A	54 (4)
C2—Fe1—C3	40.5 (3)	C11—B2—H2B	118 (3)
C13—Fe1—C3	99.1 (3)	Zr2—B2—H2B	45 (3)
C2—Fe1—C12	153.3 (2)	H2A—B2—H2B	99 (5)
C13—Fe1—C12	41.0 (3)	C11—B2—H2C	112 (5)
C3—Fe1—C12	118.1 (3)	Zr2—B2—H2C	118 (5)
C2—Fe1—C14	103.7 (3)	H2A—B2—H2C	109 (6)
C13—Fe1—C14	41.5 (3)	H2B—B2—H2C	107 (6)
C3—Fe1—C14	116.0 (3)	C5—C1—C2	103.9 (5)
C12—Fe1—C14	68.8 (3)	C5—C1—B1	131.4 (5)
C2—Fe1—C15	124.0 (3)	C2—C1—B1	124.6 (6)
C13—Fe1—C15	69.5 (3)	C5—C1—Fe1	69.1 (3)
C3—Fe1—C15	155.4 (3)	C2—C1—Fe1	66.8 (3)
C12—Fe1—C15	68.0 (3)	B1—C1—Fe1	123.7 (4)
C14—Fe1—C15	41.3 (3)	C3—C2—C1	111.4 (6)
C2—Fe1—C4	68.3 (3)	C3—C2—Fe1	70.2 (4)
C13—Fe1—C4	117.3 (3)	C1—C2—Fe1	71.2 (3)
C3—Fe1—C4	41.3 (3)	C3—C2—H2	124.3
C12—Fe1—C4	106.3 (3)	C1—C2—H2	124.3
C14—Fe1—C4	153.0 (3)	Fe1—C2—H2	125.9
C15—Fe1—C4	163.3 (3)	C2—C3—C4	107.3 (5)
C2—Fe1—C5	68.3 (3)	C2—C3—C6	126.6 (7)
C13—Fe1—C5	156.7 (3)	C4—C3—C6	125.9 (7)
C3—Fe1—C5	69.3 (2)	C2—C3—Fe1	69.3 (3)
C12—Fe1—C5	125.7 (3)	C4—C3—Fe1	70.5 (3)
C14—Fe1—C5	161.8 (3)	C6—C3—Fe1	120.8 (5)
C15—Fe1—C5	128.7 (2)	C3—C4—C5	107.5 (6)
C4—Fe1—C5	41.0 (2)	C3—C4—Fe1	68.3 (4)
C2—Fe1—C1	42.0 (2)	C5—C4—Fe1	69.6 (3)
C13—Fe1—C1	155.8 (3)	C3—C4—H4	126.2
C3—Fe1—C1	70.5 (2)	C5—C4—H4	126.2
C12—Fe1—C1	163.0 (3)	Fe1—C4—H4	127.4
C14—Fe1—C1	122.5 (3)	C1—C5—C4	110.0 (6)
C15—Fe1—C1	110.9 (2)	C1—C5—Fe1	70.2 (3)
C4—Fe1—C1	69.7 (3)	C4—C5—Fe1	69.3 (3)
C5—Fe1—C1	40.7 (3)	C1—C5—H5	125.0
C2—Fe1—C11	162.3 (3)	C4—C5—H5	125.0
C13—Fe1—C11	69.8 (3)	Fe1—C5—H5	127.1
C3—Fe1—C11	157.2 (3)	C15—C11—C12	105.5 (5)
C12—Fe1—C11	40.9 (2)	C15—C11—B2	129.9 (5)
C14—Fe1—C11	69.4 (3)	C12—C11—B2	124.5 (6)
C15—Fe1—C11	40.4 (3)	C15—C11—Fe1	68.3 (3)
C4—Fe1—C11	125.2 (3)	C12—C11—Fe1	67.9 (3)
C5—Fe1—C11	113.0 (3)	B2—C11—Fe1	127.4 (4)
C1—Fe1—C11	127.1 (2)	C13—C12—C11	110.2 (6)
C21—Zr1—C30	93.3 (2)	C13—C12—Fe1	68.9 (3)
C21—Zr1—C25	32.9 (2)	C11—C12—Fe1	71.2 (3)
C30—Zr1—C25	78.9 (2)	C13—C12—H12	124.9
C21—Zr1—C26	82.0 (2)	C11—C12—H12	124.9
C30—Zr1—C26	32.2 (2)	Fe1—C12—H12	126.7
C25—Zr1—C26	85.3 (2)	C12—C13—C14	107.2 (6)
C21—Zr1—C22	32.9 (2)	C12—C13—C8	127.8 (7)
C30—Zr1—C22	126.1 (2)	C14—C13—C8	124.8 (7)
C25—Zr1—C22	53.9 (3)	C12—C13—Fe1	70.1 (3)
C26—Zr1—C22	111.4 (2)	C14—C13—Fe1	69.9 (3)
C21—Zr1—C29	126.0 (3)	C8—C13—Fe1	121.4 (5)
C30—Zr1—C29	32.8 (2)	C13—C14—C15	107.0 (6)
C25—Zr1—C29	106.1 (2)	C13—C14—Fe1	68.6 (4)
C26—Zr1—C29	53.9 (2)	C15—C14—Fe1	69.4 (4)
C22—Zr1—C29	158.5 (3)	C13—C14—H14	126.5
C21—Zr1—C24	54.6 (2)	C15—C14—H14	126.5
C30—Zr1—C24	99.8 (2)	Fe1—C14—H14	127.0
C25—Zr1—C24	32.4 (2)	C11—C15—C14	110.0 (5)
C26—Zr1—C24	115.9 (2)	C11—C15—Fe1	71.3 (3)
C22—Zr1—C24	54.1 (2)	C14—C15—Fe1	69.2 (3)
C29—Zr1—C24	114.6 (2)	C11—C15—H15	125.0
C21—Zr1—Cl1	93.9 (2)	C14—C15—H15	125.0
C30—Zr1—Cl1	128.60 (17)	Fe1—C15—H15	126.0
C25—Zr1—Cl1	125.90 (18)	C7'—C6—C3	119.8 (9)
C26—Zr1—Cl1	99.29 (17)	C7'—C6—C7	45.7 (8)
C22—Zr1—Cl1	75.07 (18)	C3—C6—C7	114.3 (6)
C29—Zr1—Cl1	120.05 (19)	C7'—C6—H6A	64.1
C24—Zr1—Cl1	125.21 (16)	C3—C6—H6A	108.7
C21—Zr1—C23	54.5 (2)	C7—C6—H6A	108.7
C30—Zr1—C23	131.1 (2)	C7'—C6—H6B	131.1
C25—Zr1—C23	53.8 (2)	C3—C6—H6B	108.7
C26—Zr1—C23	135.6 (2)	C7—C6—H6B	108.7
C22—Zr1—C23	32.5 (2)	H6A—C6—H6B	107.6
C29—Zr1—C23	145.4 (2)	C7'—C6—H6C	107.4
C24—Zr1—C23	32.7 (2)	C3—C6—H6C	107.4
Cl1—Zr1—C23	92.96 (15)	C7—C6—H6C	66.3
C21—Zr1—C28	135.5 (2)	H6A—C6—H6C	141.8
C30—Zr1—C28	53.9 (2)	H6B—C6—H6C	47.7
C25—Zr1—C28	132.6 (2)	C7'—C6—H6D	107.4
C26—Zr1—C28	53.9 (2)	C3—C6—H6D	107.4
C22—Zr1—C28	155.6 (2)	C7—C6—H6D	137.9
C29—Zr1—C28	32.4 (2)	H6A—C6—H6D	49.7
C24—Zr1—C28	146.9 (2)	H6B—C6—H6D	60.9
Cl1—Zr1—C28	87.65 (18)	H6C—C6—H6D	106.9
C23—Zr1—C28	170.0 (2)	C8—C7—C6	122.0 (10)
C21—Zr1—C27	105.3 (2)	C8—C7—H7A	106.8
C30—Zr1—C27	53.0 (2)	C6—C7—H7A	106.8
C25—Zr1—C27	116.8 (2)	C8—C7—H7B	106.8
C26—Zr1—C27	32.0 (2)	C6—C7—H7B	106.8
C22—Zr1—C27	124.4 (2)	H6C—C7—H7B	96.1
C29—Zr1—C27	53.4 (2)	H7A—C7—H7B	106.7
C24—Zr1—C27	147.9 (2)	C6—C7'—C8	121.6 (12)
Cl1—Zr1—C27	76.04 (17)	C6—C7'—H7C	106.9
C23—Zr1—C27	156.8 (2)	C8—C7'—H7C	106.9
C28—Zr1—C27	32.4 (2)	C6—C7'—H7D	106.9
C21—Zr1—B1	138.1 (3)	C8—C7'—H7D	106.9
C30—Zr1—B1	111.4 (2)	H7C—C7'—H7D	106.7
C25—Zr1—B1	117.8 (3)	C7—C8—C13	116.7 (7)
C26—Zr1—B1	135.8 (2)	C13—C8—C7'	114.7 (7)
C22—Zr1—B1	112.7 (3)	C7—C8—H8A	108.1
C29—Zr1—B1	82.6 (2)	C13—C8—H8A	108.1
C24—Zr1—B1	87.2 (2)	C7'—C8—H8A	68.2
Cl1—Zr1—B1	95.79 (18)	C7—C8—H8B	108.1
C23—Zr1—B1	84.3 (2)	C13—C8—H8B	108.1
C28—Zr1—B1	85.7 (3)	C7'—C8—H8B	136.2
C27—Zr1—B1	116.7 (2)	H8A—C8—H8B	107.3
C21—Zr1—H1A	127 (2)	C7—C8—H8C	67.3
C30—Zr1—H1A	92 (2)	C13—C8—H8C	108.6
C25—Zr1—H1A	98 (2)	C7'—C8—H8C	108.6
C26—Zr1—H1A	122 (2)	C7—C8—H8D	133.7
C22—Zr1—H1A	117 (2)	C13—C8—H8D	108.6
C29—Zr1—H1A	70 (2)	C7'—C8—H8D	108.6
C24—Zr1—H1A	73 (2)	H8C—C8—H8D	107.6
Cl1—Zr1—H1A	122 (2)	C25—C21—C22	107.2 (6)
C23—Zr1—H1A	84 (2)	C25—C21—Zr1	74.6 (3)
C28—Zr1—H1A	87 (2)	C22—C21—Zr1	75.1 (3)
C27—Zr1—H1A	119 (2)	C25—C21—H21	126.4
B1—Zr1—H1A	26 (2)	C22—C21—H21	126.4
C21—Zr1—H1B	129 (2)	Zr1—C21—H21	116.1
C30—Zr1—H1B	137 (2)	C23—C22—C21	108.4 (6)
C25—Zr1—H1B	131 (2)	C23—C22—Zr1	74.0 (3)
C26—Zr1—H1B	143 (2)	C21—C22—Zr1	72.0 (3)
C22—Zr1—H1B	96 (2)	C23—C22—H22	125.8
C29—Zr1—H1B	105 (2)	C21—C22—H22	125.8
C24—Zr1—H1B	100 (2)	Zr1—C22—H22	120.0
Cl1—Zr1—H1B	63 (2)	C22—C23—C24	107.9 (6)
C23—Zr1—H1B	80 (2)	C22—C23—Zr1	73.5 (3)
C28—Zr1—H1B	91 (2)	C24—C23—Zr1	73.5 (3)
C27—Zr1—H1B	112 (2)	C22—C23—H23	126.0
B1—Zr1—H1B	33 (2)	C24—C23—H23	126.0
H1A—Zr1—H1B	59 (3)	Zr1—C23—H23	118.9
Cl2—Zr2—C40	79.0 (2)	C25—C24—C23	107.4 (6)
Cl2—Zr2—C34	90.3 (2)	C25—C24—Zr1	73.1 (4)
C40—Zr2—C34	100.0 (3)	C23—C24—Zr1	73.8 (4)
Cl2—Zr2—C38	132.7 (2)	C25—C24—H24	126.3
C40—Zr2—C38	53.9 (3)	C23—C24—H24	126.3
C34—Zr2—C38	100.7 (3)	Zr1—C24—H24	118.8
Cl2—Zr2—C36	84.9 (3)	C24—C25—C21	109.1 (6)
C40—Zr2—C36	33.2 (3)	C24—C25—Zr1	74.5 (4)
C34—Zr2—C36	133.0 (3)	C21—C25—Zr1	72.5 (4)
C38—Zr2—C36	54.2 (3)	C24—C25—H25	125.4
Cl2—Zr2—C39	106.42 (19)	C21—C25—H25	125.4
C40—Zr2—C39	32.4 (3)	Zr1—C25—H25	119.4
C34—Zr2—C39	82.9 (2)	C30—C26—C27	107.8 (6)
C38—Zr2—C39	32.5 (2)	C30—C26—Zr1	73.4 (3)
C36—Zr2—C39	54.4 (2)	C27—C26—Zr1	75.1 (4)
Cl2—Zr2—C37	116.3 (3)	C30—C26—H26	126.1
C40—Zr2—C37	53.6 (3)	C27—C26—H26	126.1
C34—Zr2—C37	133.0 (3)	Zr1—C26—H26	117.4
C38—Zr2—C37	32.7 (3)	C26—C27—C28	108.9 (6)
C36—Zr2—C37	32.1 (3)	C26—C27—Zr1	72.9 (4)
C39—Zr2—C37	53.7 (2)	C28—C27—Zr1	73.3 (4)
Cl2—Zr2—C31	98.5 (2)	C26—C27—H27	125.6
C40—Zr2—C31	153.5 (3)	C28—C27—H27	125.6
C34—Zr2—C31	53.5 (3)	Zr1—C27—H27	120.0
C38—Zr2—C31	125.0 (3)	C29—C28—C27	107.2 (6)
C36—Zr2—C31	172.9 (3)	C29—C28—Zr1	73.6 (4)
C39—Zr2—C31	129.6 (2)	C27—C28—Zr1	74.3 (4)
C37—Zr2—C31	143.0 (3)	C29—C28—H28	126.4
Cl2—Zr2—C33	122.7 (2)	C27—C28—H28	126.4
C40—Zr2—C33	105.8 (3)	Zr1—C28—H28	117.8
C34—Zr2—C33	32.4 (3)	C28—C29—C30	107.3 (6)
C38—Zr2—C33	79.1 (3)	C28—C29—Zr1	74.0 (4)
C36—Zr2—C33	129.7 (3)	C30—C29—Zr1	72.8 (3)
C39—Zr2—C33	76.5 (2)	C28—C29—H29	126.3
C37—Zr2—C33	110.5 (3)	C30—C29—H29	126.3
C31—Zr2—C33	53.3 (2)	Zr1—C29—H29	118.8
Cl2—Zr2—C32	129.4 (2)	C26—C30—C29	108.8 (6)
C40—Zr2—C32	135.3 (3)	C26—C30—Zr1	74.4 (4)
C34—Zr2—C32	54.0 (3)	C29—C30—Zr1	74.4 (4)
C38—Zr2—C32	92.3 (3)	C26—C30—H30	125.6
C36—Zr2—C32	145.3 (3)	C29—C30—H30	125.6
C39—Zr2—C32	103.3 (3)	Zr1—C30—H30	117.5
C37—Zr2—C32	114.3 (3)	C35—C31—C32	108.9 (7)
C31—Zr2—C32	32.7 (3)	C35—C31—Zr2	74.6 (4)
C33—Zr2—C32	32.2 (3)	C32—C31—Zr2	73.9 (4)
Cl2—Zr2—C35	76.7 (2)	C35—C31—H31	125.6
C40—Zr2—C35	124.6 (3)	C32—C31—H31	125.6
C34—Zr2—C35	32.4 (3)	Zr2—C31—H31	117.8
C38—Zr2—C35	131.2 (3)	C33—C32—C31	106.5 (7)
C36—Zr2—C35	154.9 (3)	C33—C32—Zr2	73.8 (4)
C39—Zr2—C35	114.8 (3)	C31—C32—Zr2	73.3 (4)
C37—Zr2—C35	163.7 (3)	C33—C32—H32	126.8
C31—Zr2—C35	31.9 (3)	C31—C32—H32	126.8
C33—Zr2—C35	53.3 (3)	Zr2—C32—H32	118.2
C32—Zr2—C35	53.7 (3)	C32—C33—C34	109.0 (7)
Cl2—Zr2—B2	102.28 (19)	C32—C33—Zr2	74.0 (4)
C40—Zr2—B2	125.3 (3)	C34—C33—Zr2	72.9 (4)
C34—Zr2—B2	134.3 (3)	C32—C33—H33	125.5
C38—Zr2—B2	101.9 (3)	C34—C33—H33	125.5
C36—Zr2—B2	92.1 (3)	Zr2—C33—H33	119.4
C39—Zr2—B2	132.4 (2)	C33—C34—C35	107.7 (7)
C37—Zr2—B2	79.7 (3)	C33—C34—Zr2	74.7 (4)
C31—Zr2—B2	81.1 (3)	C35—C34—Zr2	75.1 (4)
C33—Zr2—B2	117.2 (3)	C33—C34—H34	126.1
C32—Zr2—B2	86.0 (3)	C35—C34—H34	126.1
C35—Zr2—B2	108.3 (3)	Zr2—C34—H34	116.2
Cl2—Zr2—H2A	73 (2)	C31—C35—C34	107.9 (7)
C40—Zr2—H2A	116 (2)	C31—C35—Zr2	73.5 (4)
C34—Zr2—H2A	136 (2)	C34—C35—Zr2	72.5 (4)
C38—Zr2—H2A	121 (2)	C31—C35—H35	126.0
C36—Zr2—H2A	87 (2)	C34—C35—H35	126.0
C39—Zr2—H2A	141 (2)	Zr2—C35—H35	119.8
C37—Zr2—H2A	90 (2)	C37—C36—C40	106.7 (7)
C31—Zr2—H2A	88 (2)	C37—C36—Zr2	74.2 (4)
C33—Zr2—H2A	138 (2)	C40—C36—Zr2	73.0 (4)
C32—Zr2—H2A	106 (2)	C37—C36—H36	126.7
C35—Zr2—H2A	104 (2)	C40—C36—H36	126.7
B2—Zr2—H2A	29 (2)	Zr2—C36—H36	118.1
Cl2—Zr2—H2B	132 (2)	C36—C37—C38	109.3 (7)
C40—Zr2—H2B	125 (2)	C36—C37—Zr2	73.7 (4)
C34—Zr2—H2B	120 (2)	C38—C37—Zr2	73.2 (4)
C38—Zr2—H2B	80 (2)	C36—C37—H37	125.3
C36—Zr2—H2B	97 (2)	C38—C37—H37	125.3
C39—Zr2—H2B	113 (2)	Zr2—C37—H37	119.6
C37—Zr2—H2B	71 (2)	C39—C38—C37	107.6 (8)
C31—Zr2—H2B	77 (2)	C39—C38—Zr2	74.1 (4)
C33—Zr2—H2B	92 (2)	C37—C38—Zr2	74.2 (4)
C32—Zr2—H2B	66 (2)	C39—C38—H38	126.2
C35—Zr2—H2B	108 (2)	C37—C38—H38	126.2
B2—Zr2—H2B	30 (2)	Zr2—C38—H38	117.6
H2A—Zr2—H2B	60 (3)	C40—C39—C38	108.0 (6)
C1—B1—Zr1	136.9 (5)	C40—C39—Zr2	73.3 (4)
C1—B1—H1A	111 (4)	C38—C39—Zr2	73.4 (4)
Zr1—B1—H1A	56 (4)	C40—C39—H39	126.0
C1—B1—H1B	112 (3)	C38—C39—H39	126.0
Zr1—B1—H1B	52 (3)	Zr2—C39—H39	119.1
H1A—B1—H1B	108 (5)	C39—C40—C36	108.3 (7)
C1—B1—H1C	113 (4)	C39—C40—Zr2	74.2 (4)
Zr1—B1—H1C	110 (4)	C36—C40—Zr2	73.8 (4)
H1A—B1—H1C	111 (5)	C39—C40—H40	125.8
H1B—B1—H1C	101 (5)	C36—C40—H40	125.8
C11—B2—Zr2	130.5 (5)	Zr2—C40—H40	118.1
C11—B2—H2A	112 (4)		
			
C21—Zr1—B1—C1	−7.3 (9)	C22—Zr1—C24—C23	−37.0 (4)
C30—Zr1—B1—C1	−129.4 (6)	C29—Zr1—C24—C23	164.3 (4)
C25—Zr1—B1—C1	−41.0 (7)	Cl1—Zr1—C24—C23	−11.2 (4)
C26—Zr1—B1—C1	−155.2 (6)	C28—Zr1—C24—C23	161.5 (4)
C22—Zr1—B1—C1	18.9 (7)	C27—Zr1—C24—C23	−136.0 (4)
C29—Zr1—B1—C1	−145.3 (7)	B1—Zr1—C24—C23	83.8 (4)
C24—Zr1—B1—C1	−30.0 (7)	C23—C24—C25—C21	1.6 (7)
Cl1—Zr1—B1—C1	95.1 (7)	Zr1—C24—C25—C21	−64.7 (5)
C23—Zr1—B1—C1	2.7 (7)	C23—C24—C25—Zr1	66.3 (4)
C28—Zr1—B1—C1	−177.7 (7)	C22—C21—C25—C24	−2.7 (7)
C27—Zr1—B1—C1	172.4 (6)	Zr1—C21—C25—C24	66.0 (5)
Cl2—Zr2—B2—C11	−94.1 (6)	C22—C21—C25—Zr1	−68.7 (4)
C40—Zr2—B2—C11	−9.0 (8)	C21—Zr1—C25—C24	−116.4 (6)
C34—Zr2—B2—C11	163.1 (5)	C30—Zr1—C25—C24	129.2 (4)
C38—Zr2—B2—C11	44.9 (7)	C26—Zr1—C25—C24	161.1 (4)
C36—Zr2—B2—C11	−8.9 (7)	C22—Zr1—C25—C24	−78.1 (4)
C39—Zr2—B2—C11	32.1 (8)	C29—Zr1—C25—C24	110.7 (4)
C37—Zr2—B2—C11	20.8 (7)	Cl1—Zr1—C25—C24	−100.8 (4)
C31—Zr2—B2—C11	169.0 (7)	C23—Zr1—C25—C24	−37.6 (4)
C33—Zr2—B2—C11	128.6 (6)	C28—Zr1—C25—C24	132.5 (4)
C32—Zr2—B2—C11	136.5 (7)	C27—Zr1—C25—C24	167.3 (4)
C35—Zr2—B2—C11	−173.9 (6)	B1—Zr1—C25—C24	20.7 (5)
Zr1—B1—C1—C5	29.0 (11)	C30—Zr1—C25—C21	−114.4 (5)
Zr1—B1—C1—C2	−156.7 (5)	C26—Zr1—C25—C21	−82.6 (4)
Zr1—B1—C1—Fe1	119.9 (6)	C22—Zr1—C25—C21	38.2 (4)
C2—Fe1—C1—C5	−116.3 (5)	C29—Zr1—C25—C21	−132.9 (4)
C13—Fe1—C1—C5	−148.2 (6)	C24—Zr1—C25—C21	116.4 (6)
C3—Fe1—C1—C5	−80.6 (4)	Cl1—Zr1—C25—C21	15.5 (5)
C12—Fe1—C1—C5	42.5 (10)	C23—Zr1—C25—C21	78.8 (5)
C14—Fe1—C1—C5	170.4 (4)	C28—Zr1—C25—C21	−111.2 (5)
C15—Fe1—C1—C5	125.6 (4)	C27—Zr1—C25—C21	−76.3 (5)
C4—Fe1—C1—C5	−36.6 (4)	B1—Zr1—C25—C21	137.1 (4)
C11—Fe1—C1—C5	82.7 (4)	C21—Zr1—C26—C30	−109.4 (4)
C13—Fe1—C1—C2	−31.9 (8)	C25—Zr1—C26—C30	−76.4 (4)
C3—Fe1—C1—C2	35.7 (4)	C22—Zr1—C26—C30	−124.6 (4)
C12—Fe1—C1—C2	158.8 (8)	C29—Zr1—C26—C30	37.4 (4)
C14—Fe1—C1—C2	−73.3 (5)	C24—Zr1—C26—C30	−65.3 (4)
C15—Fe1—C1—C2	−118.1 (4)	Cl1—Zr1—C26—C30	157.9 (4)
C4—Fe1—C1—C2	79.7 (4)	C23—Zr1—C26—C30	−98.1 (4)
C5—Fe1—C1—C2	116.3 (5)	C28—Zr1—C26—C30	77.7 (4)
C11—Fe1—C1—C2	−161.0 (4)	C27—Zr1—C26—C30	114.2 (6)
C2—Fe1—C1—B1	117.1 (7)	B1—Zr1—C26—C30	49.6 (5)
C13—Fe1—C1—B1	85.3 (8)	C21—Zr1—C26—C27	136.4 (5)
C3—Fe1—C1—B1	152.8 (6)	C30—Zr1—C26—C27	−114.2 (6)
C12—Fe1—C1—B1	−84.0 (11)	C25—Zr1—C26—C27	169.4 (4)
C14—Fe1—C1—B1	43.9 (6)	C22—Zr1—C26—C27	121.2 (4)
C15—Fe1—C1—B1	−1.0 (6)	C29—Zr1—C26—C27	−76.8 (4)
C4—Fe1—C1—B1	−163.2 (6)	C24—Zr1—C26—C27	−179.5 (4)
C5—Fe1—C1—B1	−126.6 (6)	Cl1—Zr1—C26—C27	43.7 (4)
C11—Fe1—C1—B1	−43.8 (6)	C23—Zr1—C26—C27	147.7 (4)
C5—C1—C2—C3	0.8 (7)	C28—Zr1—C26—C27	−36.4 (4)
B1—C1—C2—C3	−174.8 (6)	B1—Zr1—C26—C27	−64.6 (5)
Fe1—C1—C2—C3	−58.8 (4)	C30—C26—C27—C28	−1.7 (7)
C5—C1—C2—Fe1	59.6 (4)	Zr1—C26—C27—C28	65.0 (4)
B1—C1—C2—Fe1	−116.0 (6)	C30—C26—C27—Zr1	−66.7 (4)
C13—Fe1—C2—C3	−71.8 (4)	C21—Zr1—C27—C26	−45.1 (5)
C12—Fe1—C2—C3	−44.2 (8)	C30—Zr1—C27—C26	37.5 (4)
C14—Fe1—C2—C3	−114.1 (4)	C25—Zr1—C27—C26	−11.9 (5)
C15—Fe1—C2—C3	−154.0 (4)	C22—Zr1—C27—C26	−74.8 (5)
C4—Fe1—C2—C3	38.8 (4)	C29—Zr1—C27—C26	78.8 (4)
C5—Fe1—C2—C3	83.1 (4)	C24—Zr1—C27—C26	0.9 (7)
C1—Fe1—C2—C3	122.2 (5)	Cl1—Zr1—C27—C26	−135.3 (4)
C11—Fe1—C2—C3	−179.0 (7)	C23—Zr1—C27—C26	−71.8 (7)
C13—Fe1—C2—C1	166.0 (4)	C28—Zr1—C27—C26	116.5 (6)
C3—Fe1—C2—C1	−122.2 (5)	B1—Zr1—C27—C26	135.1 (4)
C12—Fe1—C2—C1	−166.4 (6)	C21—Zr1—C27—C28	−161.5 (4)
C14—Fe1—C2—C1	123.7 (4)	C30—Zr1—C27—C28	−79.0 (4)
C15—Fe1—C2—C1	83.9 (4)	C25—Zr1—C27—C28	−128.3 (4)
C4—Fe1—C2—C1	−83.4 (4)	C26—Zr1—C27—C28	−116.5 (6)
C5—Fe1—C2—C1	−39.0 (4)	C22—Zr1—C27—C28	168.7 (4)
C11—Fe1—C2—C1	58.9 (9)	C29—Zr1—C27—C28	−37.7 (4)
C1—C2—C3—C4	−1.2 (7)	C24—Zr1—C27—C28	−115.6 (5)
Fe1—C2—C3—C4	−60.6 (4)	Cl1—Zr1—C27—C28	108.2 (4)
C1—C2—C3—C6	173.1 (6)	C23—Zr1—C27—C28	171.7 (5)
Fe1—C2—C3—C6	113.7 (7)	B1—Zr1—C27—C28	18.7 (5)
C1—C2—C3—Fe1	59.4 (4)	C26—C27—C28—C29	2.1 (7)
C13—Fe1—C3—C2	120.6 (4)	Zr1—C27—C28—C29	66.7 (5)
C12—Fe1—C3—C2	159.2 (4)	C26—C27—C28—Zr1	−64.7 (4)
C14—Fe1—C3—C2	80.6 (4)	C21—Zr1—C28—C29	−87.9 (5)
C15—Fe1—C3—C2	60.9 (7)	C30—Zr1—C28—C29	−37.7 (4)
C4—Fe1—C3—C2	−118.0 (5)	C25—Zr1—C28—C29	−41.6 (6)
C5—Fe1—C3—C2	−80.4 (4)	C26—Zr1—C28—C29	−77.8 (4)
C1—Fe1—C3—C2	−36.9 (4)	C22—Zr1—C28—C29	−136.8 (6)
C11—Fe1—C3—C2	179.2 (6)	C24—Zr1—C28—C29	4.9 (6)
C2—Fe1—C3—C4	118.0 (5)	Cl1—Zr1—C28—C29	178.9 (4)
C13—Fe1—C3—C4	−121.4 (4)	C23—Zr1—C28—C29	85.2 (13)
C12—Fe1—C3—C4	−82.7 (4)	C27—Zr1—C28—C29	−113.7 (6)
C14—Fe1—C3—C4	−161.4 (4)	B1—Zr1—C28—C29	82.9 (4)
C15—Fe1—C3—C4	178.9 (5)	C21—Zr1—C28—C27	25.8 (6)
C5—Fe1—C3—C4	37.6 (4)	C30—Zr1—C28—C27	76.1 (4)
C1—Fe1—C3—C4	81.1 (4)	C25—Zr1—C28—C27	72.1 (5)
C11—Fe1—C3—C4	−62.8 (7)	C26—Zr1—C28—C27	36.0 (4)
C2—Fe1—C3—C6	−121.2 (8)	C22—Zr1—C28—C27	−23.0 (8)
C13—Fe1—C3—C6	−0.6 (7)	C29—Zr1—C28—C27	113.7 (6)
C12—Fe1—C3—C6	38.1 (8)	C24—Zr1—C28—C27	118.6 (5)
C14—Fe1—C3—C6	−40.6 (8)	Cl1—Zr1—C28—C27	−67.3 (4)
C15—Fe1—C3—C6	−60.3 (10)	C23—Zr1—C28—C27	−161.0 (11)
C4—Fe1—C3—C6	120.8 (9)	B1—Zr1—C28—C27	−163.3 (4)
C5—Fe1—C3—C6	158.4 (8)	C27—C28—C29—C30	−1.6 (7)
C1—Fe1—C3—C6	−158.1 (8)	Zr1—C28—C29—C30	65.7 (5)
C11—Fe1—C3—C6	58.0 (10)	C27—C28—C29—Zr1	−67.2 (4)
C2—C3—C4—C5	1.1 (7)	C21—Zr1—C29—C28	119.9 (4)
C6—C3—C4—C5	−173.3 (6)	C30—Zr1—C29—C28	114.4 (6)
Fe1—C3—C4—C5	−58.8 (4)	C25—Zr1—C29—C28	149.4 (4)
C2—C3—C4—Fe1	59.9 (4)	C26—Zr1—C29—C28	77.8 (4)
C6—C3—C4—Fe1	−114.5 (6)	C22—Zr1—C29—C28	129.6 (6)
C2—Fe1—C4—C3	−38.1 (4)	C24—Zr1—C29—C28	−177.1 (4)
C13—Fe1—C4—C3	71.5 (5)	Cl1—Zr1—C29—C28	−1.2 (5)
C12—Fe1—C4—C3	114.3 (4)	C23—Zr1—C29—C28	−162.2 (4)
C14—Fe1—C4—C3	39.2 (8)	C27—Zr1—C29—C28	37.7 (4)
C15—Fe1—C4—C3	−178.4 (8)	B1—Zr1—C29—C28	−93.7 (4)
C5—Fe1—C4—C3	−119.6 (6)	C21—Zr1—C29—C30	5.5 (6)
C1—Fe1—C4—C3	−83.2 (4)	C25—Zr1—C29—C30	35.0 (5)
C11—Fe1—C4—C3	155.0 (4)	C26—Zr1—C29—C30	−36.6 (4)
C2—Fe1—C4—C5	81.5 (4)	C22—Zr1—C29—C30	15.3 (9)
C13—Fe1—C4—C5	−169.0 (4)	C24—Zr1—C29—C30	68.5 (5)
C3—Fe1—C4—C5	119.6 (6)	Cl1—Zr1—C29—C30	−115.6 (4)
C12—Fe1—C4—C5	−126.2 (4)	C23—Zr1—C29—C30	83.4 (5)
C14—Fe1—C4—C5	158.8 (6)	C28—Zr1—C29—C30	−114.4 (6)
C15—Fe1—C4—C5	−58.8 (11)	C27—Zr1—C29—C30	−76.7 (4)
C1—Fe1—C4—C5	36.4 (4)	B1—Zr1—C29—C30	151.9 (5)
C11—Fe1—C4—C5	−85.4 (5)	C27—C26—C30—C29	0.8 (7)
C2—C1—C5—C4	−0.1 (6)	Zr1—C26—C30—C29	−67.1 (5)
B1—C1—C5—C4	175.0 (6)	C27—C26—C30—Zr1	67.8 (4)
Fe1—C1—C5—C4	58.0 (4)	C28—C29—C30—C26	0.5 (7)
C2—C1—C5—Fe1	−58.1 (4)	Zr1—C29—C30—C26	67.0 (4)
B1—C1—C5—Fe1	117.0 (6)	C28—C29—C30—Zr1	−66.5 (5)
C3—C4—C5—C1	−0.6 (7)	C21—Zr1—C30—C26	69.3 (4)
Fe1—C4—C5—C1	−58.5 (4)	C25—Zr1—C30—C26	99.1 (4)
C3—C4—C5—Fe1	57.9 (4)	C22—Zr1—C30—C26	71.7 (5)
C2—Fe1—C5—C1	40.2 (4)	C29—Zr1—C30—C26	−115.1 (6)
C13—Fe1—C5—C1	147.0 (6)	C24—Zr1—C30—C26	124.0 (4)
C3—Fe1—C5—C1	83.8 (4)	Cl1—Zr1—C30—C26	−28.3 (5)
C12—Fe1—C5—C1	−165.9 (3)	C23—Zr1—C30—C26	113.2 (4)
C14—Fe1—C5—C1	−26.7 (10)	C28—Zr1—C30—C26	−78.0 (4)
C15—Fe1—C5—C1	−76.8 (4)	C27—Zr1—C30—C26	−37.3 (4)
C4—Fe1—C5—C1	121.6 (6)	B1—Zr1—C30—C26	−145.2 (4)
C11—Fe1—C5—C1	−120.7 (4)	C21—Zr1—C30—C29	−175.5 (5)
C2—Fe1—C5—C4	−81.4 (4)	C25—Zr1—C30—C29	−145.8 (5)
C13—Fe1—C5—C4	25.4 (9)	C26—Zr1—C30—C29	115.1 (6)
C3—Fe1—C5—C4	−37.8 (4)	C22—Zr1—C30—C29	−173.1 (4)
C12—Fe1—C5—C4	72.6 (5)	C24—Zr1—C30—C29	−120.8 (4)
C14—Fe1—C5—C4	−148.3 (8)	Cl1—Zr1—C30—C29	86.8 (5)
C15—Fe1—C5—C4	161.7 (4)	C23—Zr1—C30—C29	−131.6 (4)
C1—Fe1—C5—C4	−121.6 (6)	C28—Zr1—C30—C29	37.2 (4)
C11—Fe1—C5—C4	117.7 (4)	C27—Zr1—C30—C29	77.9 (5)
Zr2—B2—C11—C15	37.8 (10)	B1—Zr1—C30—C29	−30.1 (5)
Zr2—B2—C11—C12	−143.2 (6)	Cl2—Zr2—C31—C35	47.1 (5)
Zr2—B2—C11—Fe1	129.6 (5)	C40—Zr2—C31—C35	−35.3 (9)
C2—Fe1—C11—C15	32.7 (9)	C34—Zr2—C31—C35	−37.0 (5)
C13—Fe1—C11—C15	−81.7 (4)	C38—Zr2—C31—C35	−113.4 (5)
C3—Fe1—C11—C15	−145.6 (6)	C39—Zr2—C31—C35	−72.6 (6)
C12—Fe1—C11—C15	−118.2 (5)	C37—Zr2—C31—C35	−152.3 (5)
C14—Fe1—C11—C15	−37.3 (4)	C33—Zr2—C31—C35	−77.7 (5)
C4—Fe1—C11—C15	168.6 (3)	C32—Zr2—C31—C35	−115.4 (7)
C5—Fe1—C11—C15	123.3 (4)	B2—Zr2—C31—C35	148.3 (5)
C1—Fe1—C11—C15	78.6 (4)	Cl2—Zr2—C31—C32	162.4 (4)
C2—Fe1—C11—C12	150.9 (7)	C40—Zr2—C31—C32	80.1 (8)
C13—Fe1—C11—C12	36.6 (4)	C34—Zr2—C31—C32	78.3 (5)
C3—Fe1—C11—C12	−27.3 (8)	C38—Zr2—C31—C32	2.0 (6)
C14—Fe1—C11—C12	81.0 (4)	C39—Zr2—C31—C32	42.7 (6)
C15—Fe1—C11—C12	118.2 (5)	C37—Zr2—C31—C32	−36.9 (7)
C4—Fe1—C11—C12	−73.2 (5)	C33—Zr2—C31—C32	37.6 (5)
C5—Fe1—C11—C12	−118.5 (4)	C35—Zr2—C31—C32	115.4 (7)
C1—Fe1—C11—C12	−163.2 (4)	B2—Zr2—C31—C32	−96.4 (5)
C2—Fe1—C11—B2	−91.7 (10)	C35—C31—C32—C33	0.1 (8)
C13—Fe1—C11—B2	153.9 (6)	Zr2—C31—C32—C33	−66.9 (5)
C3—Fe1—C11—B2	90.0 (8)	C35—C31—C32—Zr2	67.0 (5)
C12—Fe1—C11—B2	117.4 (7)	Cl2—Zr2—C32—C33	90.5 (5)
C14—Fe1—C11—B2	−161.7 (6)	C40—Zr2—C32—C33	−28.1 (7)
C15—Fe1—C11—B2	−124.4 (7)	C34—Zr2—C32—C33	36.5 (5)
C4—Fe1—C11—B2	44.2 (6)	C38—Zr2—C32—C33	−65.1 (5)
C5—Fe1—C11—B2	−1.1 (6)	C36—Zr2—C32—C33	−79.1 (7)
C1—Fe1—C11—B2	−45.8 (7)	C39—Zr2—C32—C33	−34.2 (5)
C15—C11—C12—C13	0.3 (7)	C37—Zr2—C32—C33	−90.1 (5)
B2—C11—C12—C13	−179.0 (6)	C31—Zr2—C32—C33	113.3 (7)
Fe1—C11—C12—C13	−57.9 (4)	C35—Zr2—C32—C33	76.9 (6)
C15—C11—C12—Fe1	58.2 (4)	B2—Zr2—C32—C33	−166.9 (5)
B2—C11—C12—Fe1	−121.1 (6)	Cl2—Zr2—C32—C31	−22.7 (6)
C2—Fe1—C12—C13	−39.2 (8)	C40—Zr2—C32—C31	−141.3 (5)
C3—Fe1—C12—C13	−70.1 (5)	C34—Zr2—C32—C31	−76.8 (5)
C14—Fe1—C12—C13	38.9 (4)	C38—Zr2—C32—C31	−178.4 (5)
C15—Fe1—C12—C13	83.6 (4)	C36—Zr2—C32—C31	167.7 (5)
C4—Fe1—C12—C13	−113.0 (4)	C39—Zr2—C32—C31	−147.5 (5)
C5—Fe1—C12—C13	−153.7 (4)	C37—Zr2—C32—C31	156.6 (4)
C1—Fe1—C12—C13	173.4 (8)	C33—Zr2—C32—C31	−113.3 (7)
C11—Fe1—C12—C13	121.6 (6)	C35—Zr2—C32—C31	−36.4 (5)
C2—Fe1—C12—C11	−160.8 (6)	B2—Zr2—C32—C31	79.8 (5)
C13—Fe1—C12—C11	−121.6 (6)	C31—C32—C33—C34	1.6 (8)
C3—Fe1—C12—C11	168.4 (4)	Zr2—C32—C33—C34	−65.0 (5)
C14—Fe1—C12—C11	−82.7 (4)	C31—C32—C33—Zr2	66.6 (5)
C15—Fe1—C12—C11	−38.0 (4)	Cl2—Zr2—C33—C32	−113.2 (5)
C4—Fe1—C12—C11	125.4 (4)	C40—Zr2—C33—C32	159.9 (5)
C5—Fe1—C12—C11	84.7 (4)	C34—Zr2—C33—C32	−116.2 (7)
C1—Fe1—C12—C11	51.8 (10)	C38—Zr2—C33—C32	112.6 (5)
C11—C12—C13—C14	−1.1 (7)	C36—Zr2—C33—C32	133.5 (5)
Fe1—C12—C13—C14	−60.4 (4)	C39—Zr2—C33—C32	145.7 (6)
C11—C12—C13—C8	174.2 (6)	C37—Zr2—C33—C32	103.5 (5)
Fe1—C12—C13—C8	114.9 (7)	C31—Zr2—C33—C32	−38.3 (5)
C11—C12—C13—Fe1	59.3 (4)	C35—Zr2—C33—C32	−78.4 (6)
C2—Fe1—C13—C12	161.5 (4)	B2—Zr2—C33—C32	14.7 (6)
C3—Fe1—C13—C12	122.9 (4)	Cl2—Zr2—C33—C34	3.0 (5)
C14—Fe1—C13—C12	−117.9 (6)	C40—Zr2—C33—C34	−83.9 (5)
C15—Fe1—C13—C12	−79.7 (4)	C38—Zr2—C33—C34	−131.1 (5)
C4—Fe1—C13—C12	83.6 (4)	C36—Zr2—C33—C34	−110.3 (5)
C5—Fe1—C13—C12	65.1 (8)	C39—Zr2—C33—C34	−98.0 (5)
C1—Fe1—C13—C12	−175.2 (6)	C37—Zr2—C33—C34	−140.3 (5)
C11—Fe1—C13—C12	−36.5 (4)	C31—Zr2—C33—C34	77.9 (5)
C2—Fe1—C13—C14	−80.6 (5)	C32—Zr2—C33—C34	116.2 (7)
C3—Fe1—C13—C14	−119.3 (4)	C35—Zr2—C33—C34	37.8 (5)
C12—Fe1—C13—C14	117.9 (6)	B2—Zr2—C33—C34	131.0 (5)
C15—Fe1—C13—C14	38.1 (4)	C32—C33—C34—C35	−2.7 (8)
C4—Fe1—C13—C14	−158.6 (4)	Zr2—C33—C34—C35	−68.4 (5)
C5—Fe1—C13—C14	−177.0 (6)	C32—C33—C34—Zr2	65.8 (5)
C1—Fe1—C13—C14	−57.4 (8)	Cl2—Zr2—C34—C33	−177.5 (4)
C11—Fe1—C13—C14	81.4 (4)	C40—Zr2—C34—C33	103.7 (5)
C2—Fe1—C13—C8	38.6 (8)	C38—Zr2—C34—C33	48.8 (5)
C3—Fe1—C13—C8	0.0 (7)	C36—Zr2—C34—C33	99.2 (6)
C12—Fe1—C13—C8	−122.9 (8)	C39—Zr2—C34—C33	76.0 (5)
C14—Fe1—C13—C8	119.2 (8)	C37—Zr2—C34—C33	54.9 (6)
C15—Fe1—C13—C8	157.4 (7)	C31—Zr2—C34—C33	−77.1 (5)
C4—Fe1—C13—C8	−39.3 (8)	C32—Zr2—C34—C33	−36.2 (4)
C5—Fe1—C13—C8	−57.8 (11)	C35—Zr2—C34—C33	−113.6 (7)
C1—Fe1—C13—C8	61.8 (10)	B2—Zr2—C34—C33	−69.8 (6)
C11—Fe1—C13—C8	−159.4 (7)	Cl2—Zr2—C34—C35	−63.9 (5)
C12—C13—C14—C15	1.5 (7)	C40—Zr2—C34—C35	−142.8 (5)
C8—C13—C14—C15	−174.0 (6)	C38—Zr2—C34—C35	162.4 (5)
Fe1—C13—C14—C15	−59.0 (4)	C36—Zr2—C34—C35	−147.2 (5)
C12—C13—C14—Fe1	60.5 (4)	C39—Zr2—C34—C35	−170.4 (5)
C8—C13—C14—Fe1	−115.0 (6)	C37—Zr2—C34—C35	168.4 (5)
C2—Fe1—C14—C13	114.6 (4)	C31—Zr2—C34—C35	36.4 (5)
C3—Fe1—C14—C13	73.4 (5)	C33—Zr2—C34—C35	113.6 (7)
C12—Fe1—C14—C13	−38.5 (4)	C32—Zr2—C34—C35	77.3 (6)
C15—Fe1—C14—C13	−118.9 (6)	B2—Zr2—C34—C35	43.7 (7)
C4—Fe1—C14—C13	45.7 (8)	C32—C31—C35—C34	−1.7 (8)
C5—Fe1—C14—C13	176.2 (8)	Zr2—C31—C35—C34	64.9 (5)
C1—Fe1—C14—C13	155.9 (4)	C32—C31—C35—Zr2	−66.6 (5)
C11—Fe1—C14—C13	−82.4 (4)	C33—C34—C35—C31	2.7 (8)
C2—Fe1—C14—C15	−126.5 (4)	Zr2—C34—C35—C31	−65.5 (5)
C13—Fe1—C14—C15	118.9 (6)	C33—C34—C35—Zr2	68.2 (5)
C3—Fe1—C14—C15	−167.7 (4)	Cl2—Zr2—C35—C31	−131.9 (5)
C12—Fe1—C14—C15	80.4 (4)	C40—Zr2—C35—C31	161.8 (5)
C4—Fe1—C14—C15	164.6 (6)	C34—Zr2—C35—C31	115.4 (7)
C5—Fe1—C14—C15	−64.9 (10)	C38—Zr2—C35—C31	92.1 (6)
C1—Fe1—C14—C15	−85.2 (4)	C36—Zr2—C35—C31	−175.7 (6)
C11—Fe1—C14—C15	36.4 (4)	C39—Zr2—C35—C31	125.9 (5)
C12—C11—C15—C14	0.7 (6)	C37—Zr2—C35—C31	84.0 (12)
B2—C11—C15—C14	179.9 (6)	C33—Zr2—C35—C31	77.6 (5)
Fe1—C11—C15—C14	58.6 (4)	C32—Zr2—C35—C31	37.3 (5)
C12—C11—C15—Fe1	−57.9 (4)	B2—Zr2—C35—C31	−33.2 (6)
B2—C11—C15—Fe1	121.3 (6)	Cl2—Zr2—C35—C34	112.6 (5)
C13—C14—C15—C11	−1.4 (7)	C40—Zr2—C35—C34	46.3 (6)
Fe1—C14—C15—C11	−59.9 (4)	C38—Zr2—C35—C34	−23.3 (7)
C13—C14—C15—Fe1	58.5 (4)	C36—Zr2—C35—C34	68.8 (9)
C2—Fe1—C15—C11	−168.6 (3)	C39—Zr2—C35—C34	10.5 (6)
C13—Fe1—C15—C11	82.6 (4)	C37—Zr2—C35—C34	−31.5 (14)
C3—Fe1—C15—C11	148.2 (6)	C31—Zr2—C35—C34	−115.4 (7)
C12—Fe1—C15—C11	38.5 (3)	C33—Zr2—C35—C34	−37.8 (5)
C14—Fe1—C15—C11	120.9 (5)	C32—Zr2—C35—C34	−78.1 (5)
C4—Fe1—C15—C11	−34.3 (10)	B2—Zr2—C35—C34	−148.6 (5)
C5—Fe1—C15—C11	−80.3 (4)	Cl2—Zr2—C36—C37	168.9 (4)
C1—Fe1—C15—C11	−123.2 (3)	C40—Zr2—C36—C37	−113.5 (7)
C2—Fe1—C15—C14	70.5 (4)	C34—Zr2—C36—C37	−105.5 (5)
C13—Fe1—C15—C14	−38.3 (4)	C38—Zr2—C36—C37	−36.5 (4)
C3—Fe1—C15—C14	27.3 (8)	C39—Zr2—C36—C37	−76.7 (5)
C12—Fe1—C15—C14	−82.4 (4)	C33—Zr2—C36—C37	−62.0 (6)
C4—Fe1—C15—C14	−155.2 (8)	C32—Zr2—C36—C37	−19.2 (7)
C5—Fe1—C15—C14	158.7 (4)	C35—Zr2—C36—C37	−148.6 (6)
C1—Fe1—C15—C14	115.9 (4)	B2—Zr2—C36—C37	66.7 (5)
C11—Fe1—C15—C14	−120.9 (5)	Cl2—Zr2—C36—C40	−77.7 (5)
C2—C3—C6—C7'	−113.6 (13)	C34—Zr2—C36—C40	8.0 (8)
C4—C3—C6—C7'	59.7 (13)	C38—Zr2—C36—C40	77.0 (5)
Fe1—C3—C6—C7'	−27.5 (14)	C39—Zr2—C36—C40	36.7 (5)
C2—C3—C6—C7	−62.3 (11)	C37—Zr2—C36—C40	113.5 (7)
C4—C3—C6—C7	111.0 (9)	C33—Zr2—C36—C40	51.4 (7)
Fe1—C3—C6—C7	23.8 (11)	C32—Zr2—C36—C40	94.2 (7)
C7'—C6—C7—C8	48.7 (13)	C35—Zr2—C36—C40	−35.1 (10)
C3—C6—C7—C8	−60.0 (14)	B2—Zr2—C36—C40	−179.8 (6)
C3—C6—C7'—C8	58.3 (17)	C40—C36—C37—C38	−1.3 (8)
C7—C6—C7'—C8	−37.7 (10)	Zr2—C36—C37—C38	65.0 (5)
C6—C7—C8—C13	61.2 (14)	C40—C36—C37—Zr2	−66.4 (5)
C6—C7—C8—C7'	−37.3 (10)	Cl2—Zr2—C37—C36	−12.4 (5)
C12—C13—C8—C7	−115.5 (10)	C40—Zr2—C37—C36	38.6 (5)
C14—C13—C8—C7	59.0 (12)	C34—Zr2—C37—C36	105.6 (5)
Fe1—C13—C8—C7	−27.2 (12)	C38—Zr2—C37—C36	116.7 (7)
C12—C13—C8—C7'	−67.2 (11)	C39—Zr2—C37—C36	79.2 (5)
C14—C13—C8—C7'	107.3 (10)	C31—Zr2—C37—C36	−170.9 (4)
Fe1—C13—C8—C7'	21.0 (11)	C33—Zr2—C37—C36	133.5 (5)
C6—C7'—C8—C7	48.2 (13)	C32—Zr2—C37—C36	168.2 (4)
C6—C7'—C8—C13	−55.3 (17)	C35—Zr2—C37—C36	128.1 (11)
C30—Zr1—C21—C25	63.5 (4)	B2—Zr2—C37—C36	−111.1 (5)
C26—Zr1—C21—C25	93.7 (4)	Cl2—Zr2—C37—C38	−129.1 (5)
C22—Zr1—C21—C25	−113.0 (6)	C40—Zr2—C37—C38	−78.0 (6)
C29—Zr1—C21—C25	60.5 (5)	C34—Zr2—C37—C38	−11.1 (8)
C24—Zr1—C21—C25	−36.1 (4)	C36—Zr2—C37—C38	−116.7 (7)
Cl1—Zr1—C21—C25	−167.4 (4)	C39—Zr2—C37—C38	−37.5 (5)
C23—Zr1—C21—C25	−76.6 (4)	C31—Zr2—C37—C38	72.4 (7)
C28—Zr1—C21—C25	102.0 (5)	C33—Zr2—C37—C38	16.8 (6)
C27—Zr1—C21—C25	116.0 (4)	C32—Zr2—C37—C38	51.5 (6)
B1—Zr1—C21—C25	−64.3 (6)	C35—Zr2—C37—C38	11.4 (14)
C30—Zr1—C21—C22	176.5 (4)	B2—Zr2—C37—C38	132.2 (6)
C25—Zr1—C21—C22	113.0 (6)	C36—C37—C38—C39	1.8 (8)
C26—Zr1—C21—C22	−153.3 (5)	Zr2—C37—C38—C39	67.1 (5)
C29—Zr1—C21—C22	173.4 (4)	C36—C37—C38—Zr2	−65.3 (5)
C24—Zr1—C21—C22	76.9 (4)	Cl2—Zr2—C38—C39	−42.8 (5)
Cl1—Zr1—C21—C22	−54.5 (4)	C40—Zr2—C38—C39	−36.9 (4)
C23—Zr1—C21—C22	36.4 (4)	C34—Zr2—C38—C39	57.7 (5)
C28—Zr1—C21—C22	−145.1 (4)	C36—Zr2—C38—C39	−78.2 (5)
C27—Zr1—C21—C22	−131.1 (4)	C37—Zr2—C38—C39	−114.0 (8)
B1—Zr1—C21—C22	48.7 (6)	C31—Zr2—C38—C39	110.4 (5)
C25—C21—C22—C23	2.7 (7)	C33—Zr2—C38—C39	82.0 (5)
Zr1—C21—C22—C23	−65.6 (4)	C32—Zr2—C38—C39	111.5 (5)
C25—C21—C22—Zr1	68.3 (4)	C35—Zr2—C38—C39	70.2 (6)
C21—Zr1—C22—C23	116.0 (6)	B2—Zr2—C38—C39	−162.2 (5)
C30—Zr1—C22—C23	111.6 (4)	Cl2—Zr2—C38—C37	71.2 (6)
C25—Zr1—C22—C23	77.7 (4)	C40—Zr2—C38—C37	77.1 (6)
C26—Zr1—C22—C23	144.5 (4)	C34—Zr2—C38—C37	171.8 (6)
C29—Zr1—C22—C23	101.4 (7)	C36—Zr2—C38—C37	35.8 (5)
C24—Zr1—C22—C23	37.3 (4)	C39—Zr2—C38—C37	114.0 (8)
Cl1—Zr1—C22—C23	−121.2 (4)	C31—Zr2—C38—C37	−135.5 (6)
C28—Zr1—C22—C23	−167.5 (5)	C33—Zr2—C38—C37	−163.9 (6)
C27—Zr1—C22—C23	177.8 (4)	C32—Zr2—C38—C37	−134.5 (6)
B1—Zr1—C22—C23	−31.1 (5)	C35—Zr2—C38—C37	−175.8 (5)
C30—Zr1—C22—C21	−4.4 (5)	B2—Zr2—C38—C37	−48.1 (6)
C25—Zr1—C22—C21	−38.3 (4)	C37—C38—C39—C40	−1.5 (8)
C26—Zr1—C22—C21	28.5 (5)	Zr2—C38—C39—C40	65.7 (5)
C29—Zr1—C22—C21	−14.6 (9)	C37—C38—C39—Zr2	−67.2 (5)
C24—Zr1—C22—C21	−78.7 (5)	Cl2—Zr2—C39—C40	33.4 (5)
Cl1—Zr1—C22—C21	122.8 (4)	C34—Zr2—C39—C40	121.6 (5)
C23—Zr1—C22—C21	−116.0 (6)	C38—Zr2—C39—C40	−115.2 (7)
C28—Zr1—C22—C21	76.6 (7)	C36—Zr2—C39—C40	−37.6 (5)
C27—Zr1—C22—C21	61.8 (5)	C37—Zr2—C39—C40	−77.5 (5)
B1—Zr1—C22—C21	−147.1 (4)	C31—Zr2—C39—C40	149.8 (5)
C21—C22—C23—C24	−1.7 (7)	C33—Zr2—C39—C40	154.0 (5)
Zr1—C22—C23—C24	−66.0 (4)	C32—Zr2—C39—C40	171.9 (5)
C21—C22—C23—Zr1	64.3 (4)	C35—Zr2—C39—C40	116.0 (5)
C21—Zr1—C23—C22	−36.8 (4)	B2—Zr2—C39—C40	−91.3 (5)
C30—Zr1—C23—C22	−95.1 (5)	Cl2—Zr2—C39—C38	148.6 (4)
C25—Zr1—C23—C22	−77.7 (5)	C40—Zr2—C39—C38	115.2 (7)
C26—Zr1—C23—C22	−50.7 (5)	C34—Zr2—C39—C38	−123.1 (5)
C29—Zr1—C23—C22	−140.6 (5)	C36—Zr2—C39—C38	77.6 (5)
C24—Zr1—C23—C22	−115.0 (6)	C37—Zr2—C39—C38	37.7 (5)
Cl1—Zr1—C23—C22	55.8 (4)	C31—Zr2—C39—C38	−95.0 (5)
C28—Zr1—C23—C22	149.1 (12)	C33—Zr2—C39—C38	−90.8 (5)
C27—Zr1—C23—C22	−4.6 (8)	C32—Zr2—C39—C38	−72.8 (5)
B1—Zr1—C23—C22	151.4 (4)	C35—Zr2—C39—C38	−128.8 (5)
C21—Zr1—C23—C24	78.1 (4)	B2—Zr2—C39—C38	23.9 (6)
C30—Zr1—C23—C24	19.9 (5)	C38—C39—C40—C36	0.7 (8)
C25—Zr1—C23—C24	37.2 (4)	Zr2—C39—C40—C36	66.5 (5)
C26—Zr1—C23—C24	64.3 (5)	C38—C39—C40—Zr2	−65.8 (5)
C22—Zr1—C23—C24	115.0 (6)	C37—C36—C40—C39	0.4 (8)
C29—Zr1—C23—C24	−25.6 (6)	Zr2—C36—C40—C39	−66.8 (5)
Cl1—Zr1—C23—C24	170.8 (4)	C37—C36—C40—Zr2	67.2 (5)
C28—Zr1—C23—C24	−95.9 (13)	Cl2—Zr2—C40—C39	−147.5 (5)
C27—Zr1—C23—C24	110.4 (6)	C34—Zr2—C40—C39	−59.1 (5)
B1—Zr1—C23—C24	−93.6 (4)	C38—Zr2—C40—C39	36.9 (4)
C22—C23—C24—C25	0.0 (7)	C36—Zr2—C40—C39	115.0 (7)
Zr1—C23—C24—C25	−65.9 (4)	C37—Zr2—C40—C39	77.8 (5)
C22—C23—C24—Zr1	66.0 (4)	C31—Zr2—C40—C39	−60.5 (8)
C21—Zr1—C24—C25	36.7 (4)	C33—Zr2—C40—C39	−26.3 (5)
C30—Zr1—C24—C25	−50.5 (4)	C32—Zr2—C40—C39	−11.2 (6)
C26—Zr1—C24—C25	−21.1 (5)	C35—Zr2—C40—C39	−82.3 (5)
C22—Zr1—C24—C25	77.4 (5)	B2—Zr2—C40—C39	115.2 (5)
C29—Zr1—C24—C25	−81.3 (5)	Cl2—Zr2—C40—C36	97.5 (6)
Cl1—Zr1—C24—C25	103.2 (4)	C34—Zr2—C40—C36	−174.1 (6)
C23—Zr1—C24—C25	114.4 (6)	C38—Zr2—C40—C36	−78.1 (6)
C28—Zr1—C24—C25	−84.1 (6)	C39—Zr2—C40—C36	−115.0 (7)
C27—Zr1—C24—C25	−21.6 (6)	C37—Zr2—C40—C36	−37.2 (5)
B1—Zr1—C24—C25	−161.7 (4)	C31—Zr2—C40—C36	−175.5 (6)
C21—Zr1—C24—C23	−77.7 (4)	C33—Zr2—C40—C36	−141.3 (6)
C30—Zr1—C24—C23	−164.9 (4)	C32—Zr2—C40—C36	−126.2 (6)
C25—Zr1—C24—C23	−114.4 (6)	C35—Zr2—C40—C36	162.7 (5)
C26—Zr1—C24—C23	−135.5 (4)	B2—Zr2—C40—C36	0.2 (7)

### Hydrogen-bond geometry (Å, º)

**Table:**

*D*—H···*A*	*D*—H	H···*A*	*D*···*A*	*D*—H···*A*
C25—H25···Cl1^i^	0.95	2.75	3.466 (7)	133
C28—H28···Cl1^ii^	0.95	2.80	3.496 (8)	131
C30—H30···Cl2	0.95	2.82	3.623 (7)	143

Symmetry codes: (i) −*x*+2, *y*+1/2, −*z*+3/2; (ii) −*x*+2, −*y*, −*z*+1.
